# Efficacy and safety of nab-paclitaxel plus platinum in non-small cell lung cancer: a meta-analysis

**DOI:** 10.3389/fmed.2023.1139248

**Published:** 2023-07-24

**Authors:** Tianying Tan, Shuangshuang Li, Wenting Hu, Tinghui Yue, Qi Zeng, Xingling Zeng, Xiaochao Chen, Xiangdong Zhao, Tianbao Xiao

**Affiliations:** ^1^College of Clinical Medicine, Guizhou University of Traditional Chinese Medicine, Guiyang, Guizhou, China; ^2^School of Basic Medicine, Guizhou University of Traditional Chinese Medicine, Guiyang, Guizhou, China; ^3^Clinical Medicine College, Chengdu University of Traditional Chinese Medicine, Chengdu, China; ^4^Colorectal and Anal Surgery, Chengdu Anorectal Hospital, Chengdu, Sichuan, China; ^5^Colorectal and Anal Surgery, Shenzhen Traditional Chinese Medicine Hospital, Shenzhen, China; ^6^Colorectal and Anal Surgery, The First Affiliated Hospital of Guizhou University of Traditional Chinese Medicine, Guiyang, Guizhou, China

**Keywords:** non-small cell lung cancer, nab-paclitaxel, chemotherapy, efficacy, safety, meta-analysis

## Abstract

**Purpose:**

This meta-analysis was exerted in assessing the anticancer efficacy and safety of nab-paclitaxel (nab-P) when combined with platinum compound agents for therapy in patients with non-small cell lung cancer (NSCLC).

**Method:**

We systematically searched the following seven electronic databases: PubMed, Cochrane Library, Web of Science, Embase, CNKI, Wan Fang, and China Science and Technology Journal Data. Randomized comparative clinical [randomized controlled clinical trial (RCT)] studies on nab-P plus platinum and carboplatin or cisplatin in combination with conventional chemotherapy agents or traditional paclitaxel were searched.

**Results:**

A total of 19 RCT studies involving 6,011 patients were analyzed. The primary outcome includes the overall response rate (ORR), overall survival (OS), and progression-free survival (PFS). The secondary outcome includes adverse events (AEs). Nab-P combined with platinum (carboplatin/cisplatin) had a better ORR [odds ratio (OR) = 1.66, 95% confidence interval (CI) (1.34, 2.05), *p* < 0.001] and improved PFS [hazard ratio (HR) = 0.84, 95% CI: (0.74, 0.94), *p* = 0.01] and OS [HR = 0.86, 95% CI: (0.78, 0.96), *p* = 0.008] in NSCLC patients. ORR [OR = 2.18, 95% CI: (1.07, 4.43)], PFS [HR = 0.62, 95% CI: (0.40, 0.97)], and OS [HR = 0.63, 95% CI: (0.49, 0.81)] were significantly improved among patients aged >70 years, and ORR [OR = 1.80, 95% CI: (1.20, 2.70)] and PFS [HR = 0.74, 95% CI: (0.56, 0.97)] were significantly elevated with SCC rate ≥65% in NSCLC patients (all *p* > 0.05). Among the adverse effects, the prevalence of neutropenia, neuralgia, and arthralgia/myalgia (≥ grade 3) compared to that of the control group. On the other hand, the prevalence of anemia and thrombocytopenia was higher in the nab-P plus platinum (carboplatin/cisplatin) compared to that of controls. It is worth noting that fatigue did not show statistical significance.

**Conclusion:**

Nab-P in combination with carboplatin/cisplatin regimen improves efficacy and tolerability in patients with NSCLC.

**Systematic review registration:**

http://www.crd.york.ac.uk/PROSPERO/, identifier: CRD42022288499.

## 1. Introduction

Lung cancer, one of the most common cancers, ranked second in new cancer cases (11.4%, 2,206,771) and first in new deaths (18.0%, 1,796,144) in 2020 (1). Based on the cellular origin, lung cancer can be classified into two major categories, namely, small cell type and non-small cell type (2), with non-small cell lung cancer (NSCLC) constituting ~85% of the lung cancer (3). NSCLC is categorized into three subtypes, namely, lung adenocarcinoma, squamous cell lung cancer, and large cell carcinoma (4). NSCLC patients with a survival rate of 5 years are only ~15%, as the majority of patients with NSCLC are unresectable or metastatic at diagnosis (5) and are unsuitable for excision surgery, but they instead ought to undergo aggressive systemic therapy (consisting of chemotherapy, targeted therapy, or a combination of both) to achieve a good benefit. Nowadays, nab-P plus platinum compounds have been recommended as chemotherapy regimens to cure advanced NSCLC (6, 7).

Nab-P, a paclitaxel of solvent micelles-free, was revealed superior response rates and tolerability than solvent-based paclitaxel treatment regimens with patients in NSCLC and MBC (8). Albumin, as a natural transport carrier, is a general carrier for drug delivery into tumors due to features of abundance in blood and long half-life (9), with high accumulation in tumor tissues. It seems that promoting albumin binding to albumin-specific receptor mediated transport mechanisms to access tumors by enhanced permeation and retention (EPR) effect (10, 11). Thus, nab-P could reach the tumor microenvironment more effectively and accumulate in the tumor. In the study (10), nab-paclitaxel (nab-P) and sb-paclitaxel were radiolabeling then quantified the paclitaxel reaching tumors presenting that tumors have absorbed one-third more nab-P. This likewise illustrated that nab-P takes advantage of albumin mechanisms so that it could reach tumors more advantageously and consequently inhibit tumor growth.

The NCCN guidelines suggest that albumin-bound paclitaxel combined with platinum was used as standard treatment (6). A stage III/IV squamous NSCLC, clinical trial, indicated the beneficial effects of nab-P plus platinum compounds as the first-line chemotherapy for patients who have commonly used advanced NSCLC (12). In addition, in a clinical trial, among patients with NSCLC aged ≥60 years, nab-P plus carboplatin (nab-P + C) considerably increased overall response rate (ORR; 34 vs. 25.6%) and prolonged overall survival (OS; 13.8 vs. 11.0 months) compared with solvent-based paclitaxel in combination with carboplatin; however, it did not significantly show progression-free survival (PFS; 6.9 vs. 5.7 months). Among adverse effects, the maximum number of neutropenia incidence (≥ grade 3) was lower in nab-P + C than sb-paclitaxel plus carboplatin (134 vs. 152) and the incidence of anemia (74 vs. 16) or thrombocytopenia (45 vs. 20) is slightly higher in nab-P + C (13). However, single studies generally have heterogeneity and risk of bias, and the accuracy of study results may be limited. We, therefore, systematically assessed the therapeutic efficacy and tolerability of nab-P plus platinum using meta-analysis in accordance with published clinical trial studies and standard methods.

## 2. Materials and methods

### 2.1. Literature search

We searched, using the keywords “Carcinoma, Non-Small-Cell Lung” and “Albumin-Bound Paclitaxel,” PubMed, Cochrane Library, Web of Science, Embase, CNKI, Wan Fang, and China Science and Technology Journal Data databases from 1 January 2012 until the end of May 2022.

### 2.2. Eligibility criteria

The inclusion criteria were as follows: (1) the study category was a clinical trial or prospective study; (2) the study was a randomized controlled trial; (3) the study population was patients with histologically or pathologically confirmed NSCLC; (4) the study involved different intervention drugs, using albumin-bound paclitaxel combined with platinum chemotherapy in the trial group and platinum combined with other drugs in the control group or traditional paclitaxel; and (5) outcomes reported post-intervention as ORR, PFS, OS, adverse event (AE) outcomes grade ≥3.

The exclusion criteria were as follows: (1) articles in which the required information was not available and (2) article types, namely, retrospective study, case report, meta-analysis, review, and animal studies.

### 2.3. Research quality assessment

We checked the quality of each included study using Cochrane and evaluated the risk of bias using Review Manager 5.4.

#### 2.3.1. Data extraction

After screening by two authors, the information extracted for the selected articles included the first author's name and year of publication; patient number, gender, and age median; squamous cell carcinoma (SCC)/non-SCC; and style of study (Table 1). Table 2 includes treatment regimen per group; number of samples studied; treatment duration; primary outcome points including ORR, PFS, and OS; and subsidiary outcome points including the number of AEs ≥ grade 3 (in the case of a disagreement, a consensus was reached by conferring with the third author).

**Table 1:** Characteristics of the included comparative studies.

**References**	**Years**	**Age median (range)**		**Gender (male/female)**		**Design**	**SCC/non-SCC**	
		**C**	**E**	**C**	**E**		**C**	**E**
Langer et al. (A) (13)	2015	67 (60–84)	66 (60–NR)	197/84	192/73	RCT	110/171	106/159
Langer et al. (B) (13)		72 (70–84)	72 (70–81)	58/24	55/19	RCT	30/52	25/49
Langer et al. (14)	2015	57 (24–77)	57 (28–78)	231/60	233/56	RCT	112/179	125/164
Socinski et al. (15)	2013	59 (34–84)	59 (28–81)	199/22	205/24	RCT	221/0	229/0
Socinski et al. (16)	2013	59 (24–69)	58 (28–69)	339/110	337/110	RCT	191/258	194/253
Socinski et al. (17)	2012	60 (24–84)	60 (28–81)	397/134	392/129	RCT	221/310	229/292
Satouchi et al. (18)	2013	64 (36–77)	65 (37–79)	50/25	51/23	RCT	7/68	10/64
Hirsh et al. (19)	2016	60 (24–84)	59 (28–80)	377/70	366/69	RCT	213/288	220/270
Wang et al. (20)	2019	60 (39–76)	58 (41–79)	57/7	56/4	RCT	64/0	60/0
Xie and Wang (21)	2021	52.3 (37–77)	52.5 (38–78)	21/9	20/10	RCT	NR	
Gao and Zhu (22)	2014	NR		18/13	17/14	RCT	8/23	8/23
Zhu et al. (23)	2018	59 (43–74)	60 (39–78)	35/25	40/20	RCT	60/0	62/0
Chen and Sun (24)	2022	57.8 (43–75)	58.0 (45–77)	40/11	38/13	RCT	23/28	24/27
Qin et al. (25)	2019	56 (36–72)	55.4 (39–71)	34/9	36/5	RCT	9/34	10/31
Wang et al. (26)	2021	60 (41–74)	63 (38–74)	107/13	112/7	RCT	120/0	119/0
Kogure et al. (27)	2021	76 (73–78)	76 (73–80)	85/12	82/13	RCT	98/0	98/0
Su (28)	2022	51.2 (36–75)	53.1 (37–78)	18/10	20/8	RCT	NR	
Wang et al. (29)	2022	53.6 (35–79)	55.3 (39–80)	25/15	28/12	RCT	NR	
Cao and Fang (30)	2020	70.6 (58–81)	69.3 (57–82)	20/8	21/7	RCT	10/18	11/17

C, control; E, experimental; SCC, Squamous cell carcinoma; NR, not reported.

**Table 2:** Interventions and outcome indicators of the included studies.

**References**	**Stage**	**Number**		**Interventions**		**Duration**	**Indicators**
		**C**	**E**	**C**	**E**		
Langer et al. (A) (13)	IIIb IV	281	265	d1 d8 d15 sb-paclitaxel 200 mg/m^2^ + carboplatin (AUC) 6 mg × min/mL	d1 d8 d15 nab-P 100 mg/m^2^ + carboplatin (AUC) 6 mg × min/mL	q3w-unacceptabl	(1)–(10)
Langer et al. (B) (13)	IIIb IV	82	74	d1 d8 d15 sb-paclitaxel 200 mg/m^2^ + carboplatin (AUC) 6 mg × min/mL	d1 d8 d15 nab-P 100 mg/m^2^ + carboplatin (AUC) 6 mg × min/mL	q3w-unacceptable	
Langer et al. (14)	IIIb IV	291	289	d1 d8 d15 sb-paclitaxel 200 mg/m^2^ + carboplatin (AUC) 6 mg × min/mL	d1 d8 d15 nab-P 100 mg/m^2^ + carboplatin (AUC) 6 mg × min/mL	q3w-unacceptable	
Socinski et al. (15)	IIIb IV	221	229	d1 d8 d15 sb-paclitaxel 200 mg/m^2^ + carboplatin (AUC) 6 mg × min/mL	d1 d8 d15 nab-P 100 mg/m^2^ + carboplatin (AUC) 6 mg × min/mL	q3w-unacceptable	
Socinski et al. (16)	IIIb IV	449	447	d1 d8 d15 sb-paclitaxel 200 mg/m^2^ + carboplatin (AUC) 6 mg × min/mL	d1 d8 d15 nab-P 100 mg/m^2^ + carboplatin (AUC) 6 mg × min/mL	q3w-unacceptable	
Socinski et al. (17)	IIIb IV	531	521	d1 d8 d15 sb-paclitaxel 200 mg/m^2^ + carboplatin (AUC) 6 mg × min/mL	d1 d8 d15 nab-P 100 mg/m^2^ + carboplatin (AUC) 6 mg × min/mL	q3w-unacceptable	
Satouchi et al. (18)	IIIb IV	75	74	d1 d8 d15 sb-paclitaxel 200 mg/m^2^ + carboplatin (AUC) 6 mg × min/mL	d1 d8 d15 nab-P 100 mg/m^2^ + carboplatin (AUC) 6 mg × min/mL	q3w-unacceptable	
Hirsh et al. (19)	IIIb IV	501	490	d1 d8 d15 sb-paclitaxel 200 mg/m^2^ + carboplatin (AUC) 6 mg × min/mL	d1 d8 d15 nab-P 100 mg/m^2^ + carboplatin (AUC) 6 mg × min/mL	q3w-unacceptable	
Wang et al. (20)	IIIa IIIb IV	64	60	d1 d8 gemcitabine 1,250 mg/m^2^ + d1 carboplatin AUC 5 mg × min/mL	d1 d8 nab-P 135mg/m^2^ + d1 carboplatin (AUC) 5 mg × min/mL	q3w × 6	(1)–(6) (8) (9)
Xie and Wang (21)	IIIb IV	30	30	d1 d8 gemcitabine 1,000 mg/m^2^ + cisplatin 30 mg	d1 d8 nab-P 125 mg/m^2^ + cisplatin 30 mg	q3w × 3	(1) (8)
Gao and Zhu (22)	IIIb IV	31	31	d1 d8 paclitaxel lipsome 135–175 mg/m^2^ + d1 cisplatin 60–75 mg/m^2^	d1 d8 nab-P 130 mg/m^2^ + d1 cisplatin 60–75 mg/m^2^L	q3w × 2	(1) (2) (6) (8)
Zhu et al. (23)	IIIb IV	60	62	d1 d8 gemcitabine 1,250 mg/m^2^ + cisplatin 75 mg/m^2^	d1 d8 nab-P 135 mg/m^2^ + cisplatin 75 mg/m^2^	q3w × 2	(1) (4) (6)
Chen and Sun (24)	IIIb-IV	51	51	d1 paclitaxel 175 mg/m^2^ + d1 carboplatin (AUC) 6 mg × min/mL	d1 d8 nab-P 130 mg/m^2^ + d1 carboplatin (AUC) 6 mg × min/mL	q3w	(1) (4)–(10)
Qin et al. (25)	IIIb IV	43	41	d1 d8 gemcitabine 1,000 mg/m^2^ + d1 cisplatin 75 mg/m^2^	d1 d8 d15 nab-P 135 mg/m^2^ + d1 cisplatin 75 mg/m^2^	q3w × 6	(1)–(8) (10)
Wang et al. (26)	IIIb IV	120	119	d1 tislelizumab 200 mg + paclitaxel 175 mg/m^2^ + carboplatin AUC 5	d1 tislelizumab 200 mg + d1 d8 d15 nab-P 100 mg/m^2^ + carboplatin AUC 5	q3w × 4–6	(1)–(6) (8) (9)
Kogure et al. (27)	IIIb IV recurrence	98	98	d1 docetaxel 60 mg/m^2^	d1 d8 d15 nab-P 100 mg/m^2^ + carboplatin (AUC) 6 mg × min/mL	q3w-unacceptable	(1)–(7) (9) (10)
Su (28)	IIIb IV	28	28	d1 d8 gemcitabine 1,000 mg/m^2^ + d1 cisplatin 75 mg/m^2^ 2	d1 d8 nab-P 125 mg/m^2^ + d1 cisplatin 75 mg/m^2^	q3w × 3	(1)
Wang et al. (29)	IIIb IV	40	40	d1 d8 gemcitabine 1,000 mg/m^2^ + d1 d8 cisplatin 75 mg/m^2^	d1 d8 nab-P 125 mg/m^2^ + d1 d8 cisplatin 75 mg/m^2^	q3w × 3	(1)
Cao and Fang (30)	IIIb IV	28	28	d1 d8 gemcitabine 1,000 mg/m^2^ + d1 d8 cisplatin 60 mg/m^2^	d1 d8 nab-P 100 mg/m^2^ + d1 d8 cisplatin 75 mg/m^2^	q3w × 2	(1)

ORR, overall response rate; PFS, progression- free survival; OS, overall survival; C, control; E, experimental; SCC, Squamous cell carcinoma; RR, rate ratio; HR, hazard ratio; 95% CI, 95% confidence interval. nab-P, nab-P; C, carboplatin; Cis, cisplatin; Gem, gemcitabine; sb-P, solvent-based paclitaxel; Pa, paclitaxel; P-Lip, paclitaxel liposome.

(1) ORR; (2) PFS; (3) OS; (4) Neutropenia; (5) Anemia; (6) Thrombocytopenia; (7) Neuropathy; (8) Gastrointestinal reactions; (9) Arthralgia/Myalgia; (10) Fatigue.

#### 2.3.2. Statistical analysis

Meta-analysis was compared with the pooled results of hazard ratio (HR) with 95% confidence interval (CI) of the primary outcome (ORR, OS, and PFS) and the number of AEs (≥ grade 3), using the STATA14.0 software. The heterogeneity present in the studies was estimated by Cochrane's *Q*-test and *I*^2^ statistics test, and the data were analyzed to use a random-effects model if the *I*^2^ was >50% or heterogeneity *p* was < 0.1. In the opposite case, a fixed-effects model was used. Moreover, *p*-values > 0.05 were not recognized as statistically significant. A sensitivity analysis was generated using Comprehensive Meta-Analysis V3 to judge the stability of the pooled results. Publication bias was examined by contour-enhanced funnel plots and Begg's and Egger's tests. Publication bias existed when there was asymmetry in the funnel plot or *p* < 0.05 in Begg's and Egger's tests.

#### 2.3.3. Evidence grading

The Grading of Recommendations, Assessment, Development, and Evaluations (GRADE) guideline suggested a tool that estimated the evidence quality for 10 outcomes (ORR, PFS, OS, neutropenia, anemia, thrombocytopenia, neuropathic pain, gastrointestinal reactions, joint myalgia, and fatigue).

## 3. Results

**Figure 1:**
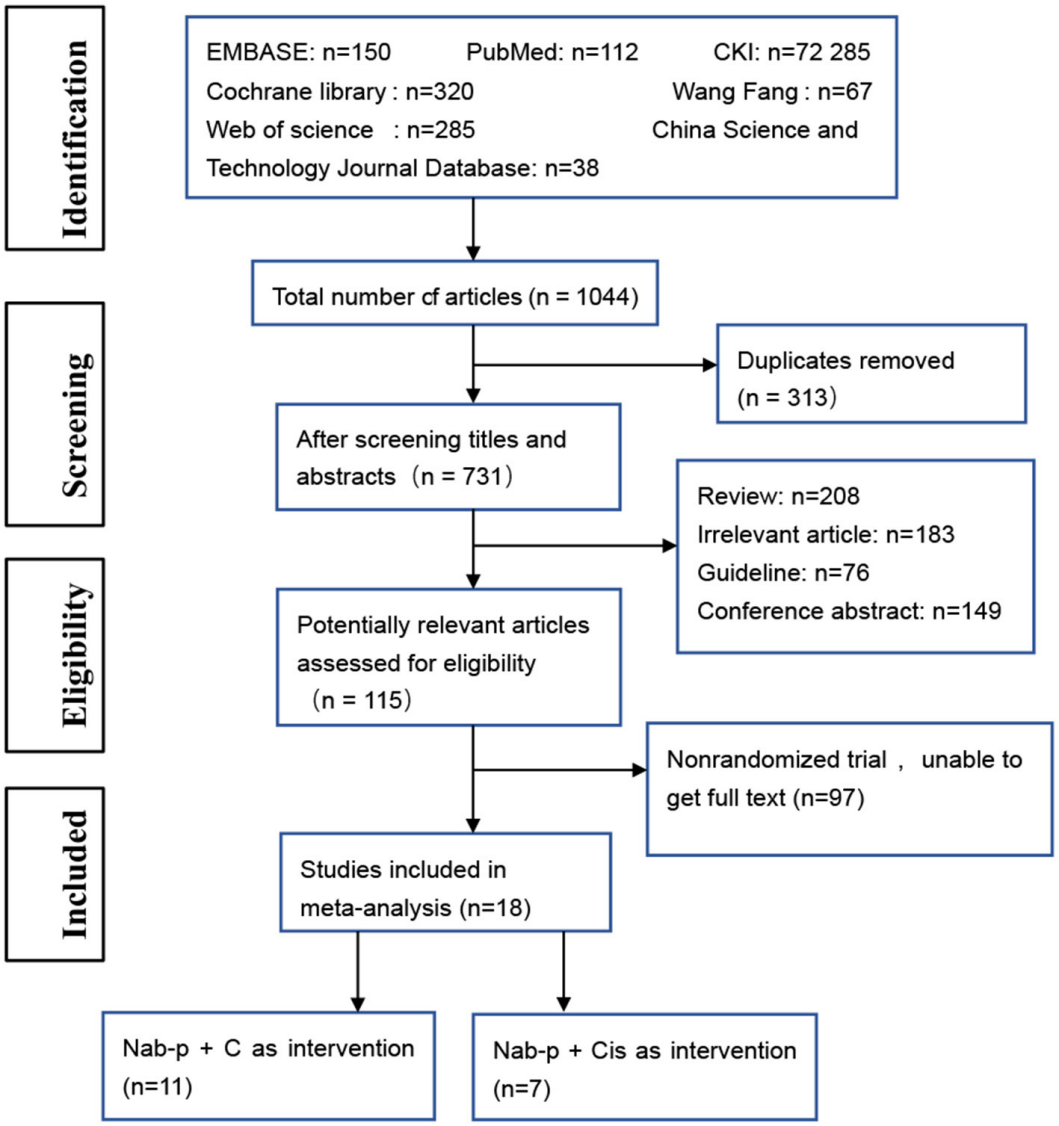
The flow diagram of the study selection process. nab-P + C, nab-p combined with carboplatin; nab-P + Cis, nab-p combined with cisplatin.

We searched 1,044 studies after screening the electronic databases. Excluding 1,026 studies based on the titles and abstracts, we finally obtained 18 studies for random trial studies that conformed to the inclusion and exclusion criteria (Figure 1). An included original study (13) grouped the data pooled according to age and was considered as two articles at inclusion. Therefore, the total number of included studies was 19. The total sample size of meta-analysis was 6,011 cases. The general characteristics of the patients are presented in Table 1. To enable research, we aggregated the drug interventions referred to in the included reports as shown in Table 2.

**Figure 2:**
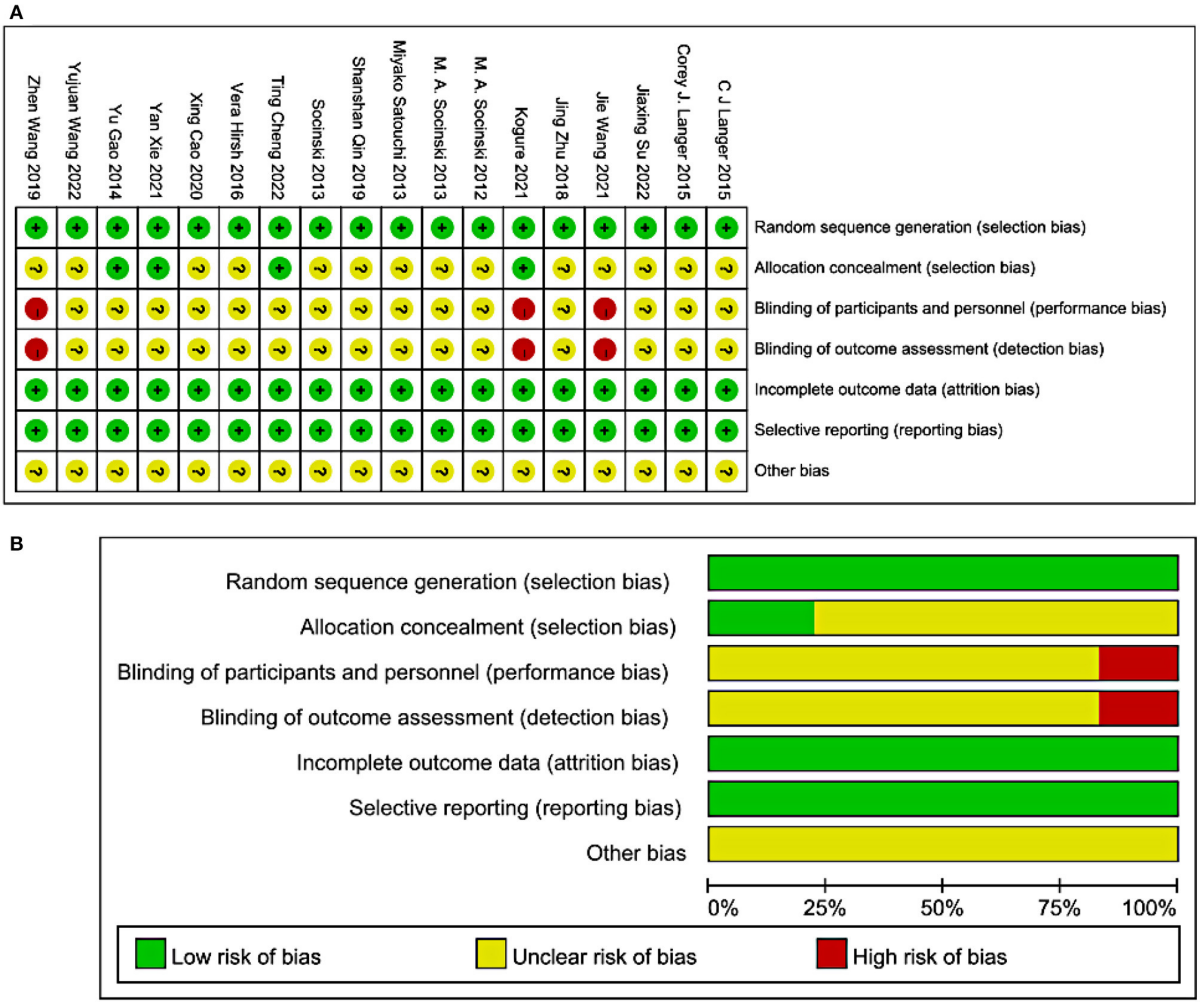
Methodological quality graph and summary of the included studies: **(A)** Risk of bias summary; +, low risk of bias; –, high risk of bias; ?, unclear risk of bias; **(B)** Risk of bias graph.

The methodological quality graph and summary of all included studies are shown in Figures 2A, B. The included articles do not address the allocation concealment. Furthermore, none of the studies had or illustrated double-blind procedures.

### 3.1. Efficacy analysis

#### 3.1.1. ORR

**Figure 3:**
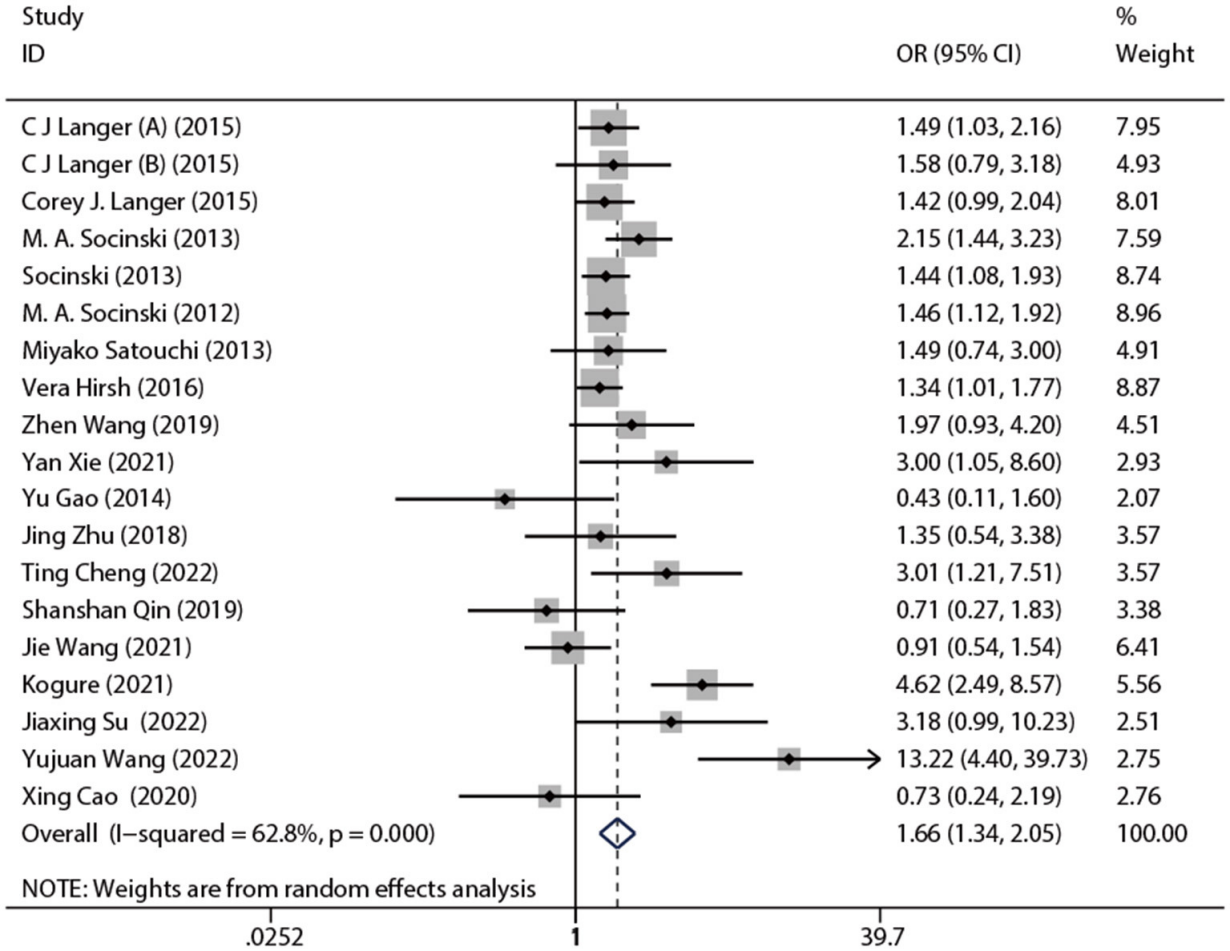
Forest plot of ORR of nab-P + C/Cis arm vs. control arm. The random model was used for ORR.

The included 19 studies reported ORR. The pooled ORR was 1.66 [95% CI: (1.34, 2.05), *p* < 0.001]. The results of the meta-analysis indicated evidence of higher ORR after nab-P + C/Cis therapy compared with carboplatin/cisplatin in combination with conventional chemotherapy agents or traditional paclitaxel (Figure 3), with significant heterogeneity (*I*^2^ = 62.8%, *p* = 0.469) than control. Therefore, a random-effects model was employed for analysis. The subgroup analysis forest plot of ORR is generated in Table 3. The nab-P + C group included 12 studies and the nab-P + Cis group included 7 studies, and significant heterogeneity appeared in nab-P + C (*I*^2^ = 51.5%, *p* = 0.019) and nab-P + Cis (*I*^2^ = 76.5%, *p* < 0.001) groups. No distinct difference was observed between nab-P + C [OR: 1.62, 95% CI: (1.34, 1.94), *p* < 0.001] ORR and overall ORR. Additionally, the nab-P + Cis [OR: 1.69, 95% CI: (0.73, 3.91), *p* = 0.220] subgroup did not arrive at statistical significance. Among the subgroup analyses by age median-based, ORR was significantly elevated in the subgroup of patients with NSCLC aged >70 years [OR: 2.18, 95% CI: (1.07, 4.43), *p* = 0.031] than the subgroup of patients aged ≤ 60 years [OR: 1.85, 95% CI: (1.39, 2.46), *p* < 0.001], and significant heterogeneity between subgroups of both age > 70 years (*I*^2^ = 79.7%, *p* = 0.007) and ≤ 60 years (*I*^2^ = 62.2%, *p* = 0.003) was showed. The SCC rate was ≥65% of ORR [OR: 1.80, 95% CI: (1.20, 2.70), *p* = 0.004] elevated even more significantly than 35–65% [OR: 1.43, 95% CI: (1.22, 1.67), *p* < 0.001]. Significant heterogeneity in SCC rate was ≥65% (*I*^2^ = 72.9%, *p* = 0.002).

**Table 3:** Subgroup analysis of overall response rate, progression-free survival and overall survival.

	**Number of studies**	**95% CI**	***I* ^2^**	***p* heterogeneity**	***p*-value**
**Subgroup of ORR**					
Overall effect on ORR	19	1.66 (1.34, 2.05)	62.8%	< 0.001	< 0.001
**Intervention**					
nab-P + C	12	1.62 (1.34, 1.94)	51.5%	0.019	< 0.001
nab-P + Cis	7	1.69 (0.73, 3.91)	76.5%	< 0.001	0.220
**Median age**					
≤ 60	11	1.85 (1.39, 2.46)	62.2%	0.003	< 0.001
60–70	4	1.26 (0.95, 1.67)	19.4%	0.293	0.114
>70	3	2.18 (1.07, 4.43)	79.7%	0.007	0.031
**SCC rate (%)**					
≤ 35%	4	1.09 (0.64, 1.86)	33.4%	0.212	0.793
35–65%	6	1.43 (1.22, 1.67)	0.0%	0.514	< 0.001
≥65%	6	1.80 (1.20, 2.70)	72.9%	0.002	0.004
**Subgroup of PFS**					
Overall effect on PFS	13	0.84 (0.74, 0.94)	51.4%	0.010	0.003
**Intervention**					
nab-P + C	11	0.83 (0.74, 0.94)	57.9%	0.008	0.003
nab-P + Cis	2	0.87 (0.44, 1.72)	55.3%	0.135	0.696
**Median age**					
≤ 60	6	0.93 (0.85, 1.02)	0.0%	0.680	0.077
60–70	3	0.89 (0.77, 1.03)	0.0%	0.963	0.131
>70	3	0.62 (0.40, 0.97)	81.3%	0.005	0.038
**SCC rate (%)**					
≤ 35%	4	0.83 (0.63, 1.08)	3.5%	0.375	0.170
35–65%	4	0.92 (0.84, 1.02)	0.0%	0.597	0.102
≥65%	5	0.74 (0.56, 0.97)	77.1%	0.002	0.030
**Subgroup of OS**					
Overall effect on OS	11	0.86 (0.78, 0.96)	56.5%	0.011	0.008
**Intervention**					
nab-P + C	10	0.86 (0.77, 0.96)	60.5%	0.007	0.008
nab-P + Cis	1	1.02 (0.58, 1.79)	-	-	0.945
**Median age**					
≤ 60	6	0.96 (0.89, 1.05)	0.0%	0.965	0.365
60–70	2	0.92 (0.80, 1.06)	0.0%	0.970	0.253
>70	3	0.63 (0.49, 0.81)	55.5%	0.106	< 0.001
**SCC rate (%)**					
≤ 35%	3	0.80 (0.56, 1.13)	43.9%	0.168	0.208
35–65%	4	0.93 (0.83, 1.04)	39.3%	0.176	0.212
≥65%	4	0.80 (0.63, 1.02)	73.9%	0.009	0.070

#### 3.1.2. PFS and OS

**Figure 4:**
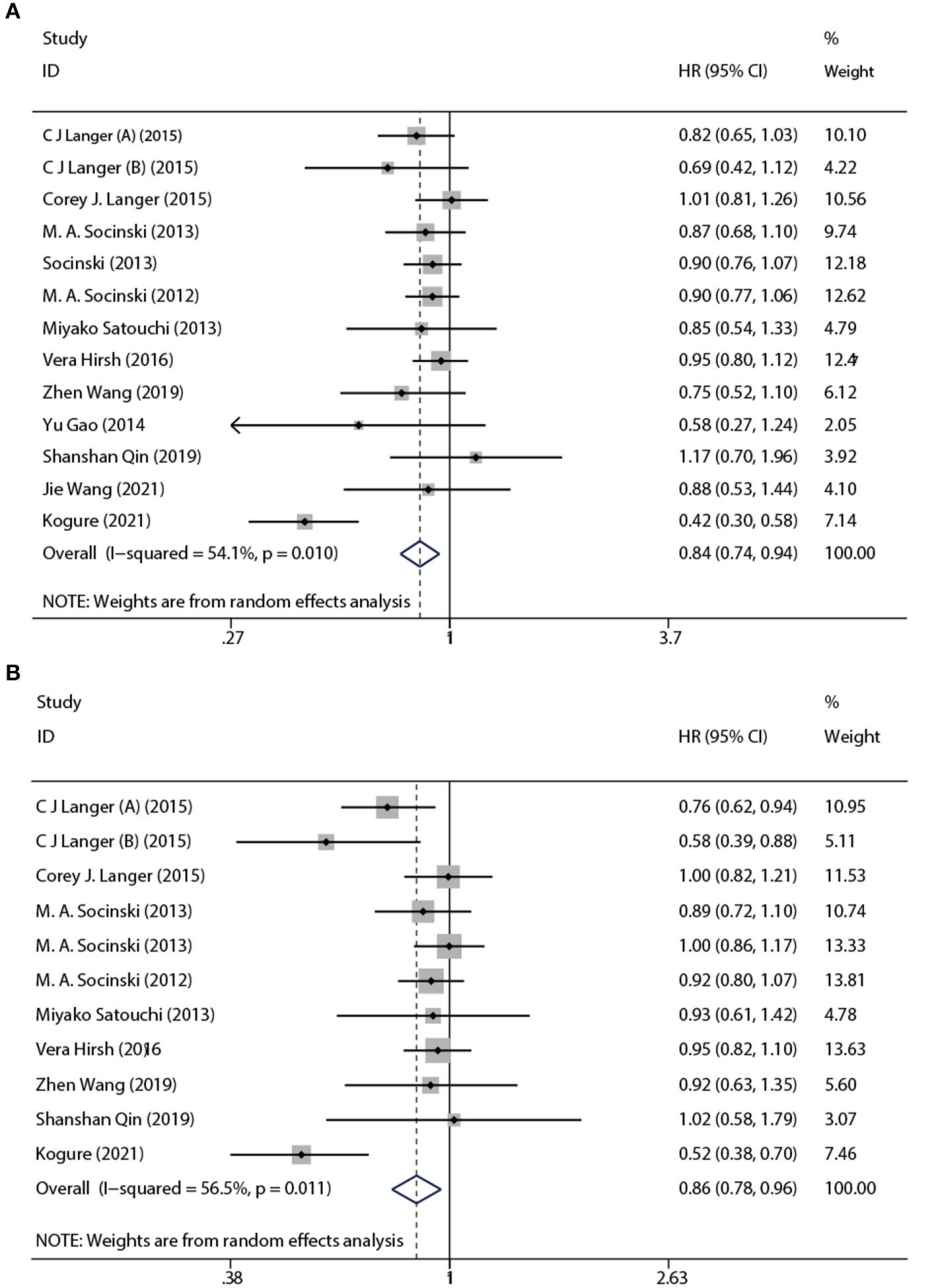
Forest plots of HR for **(A)** PFS and **(B)** OS.

**Figure 5:**
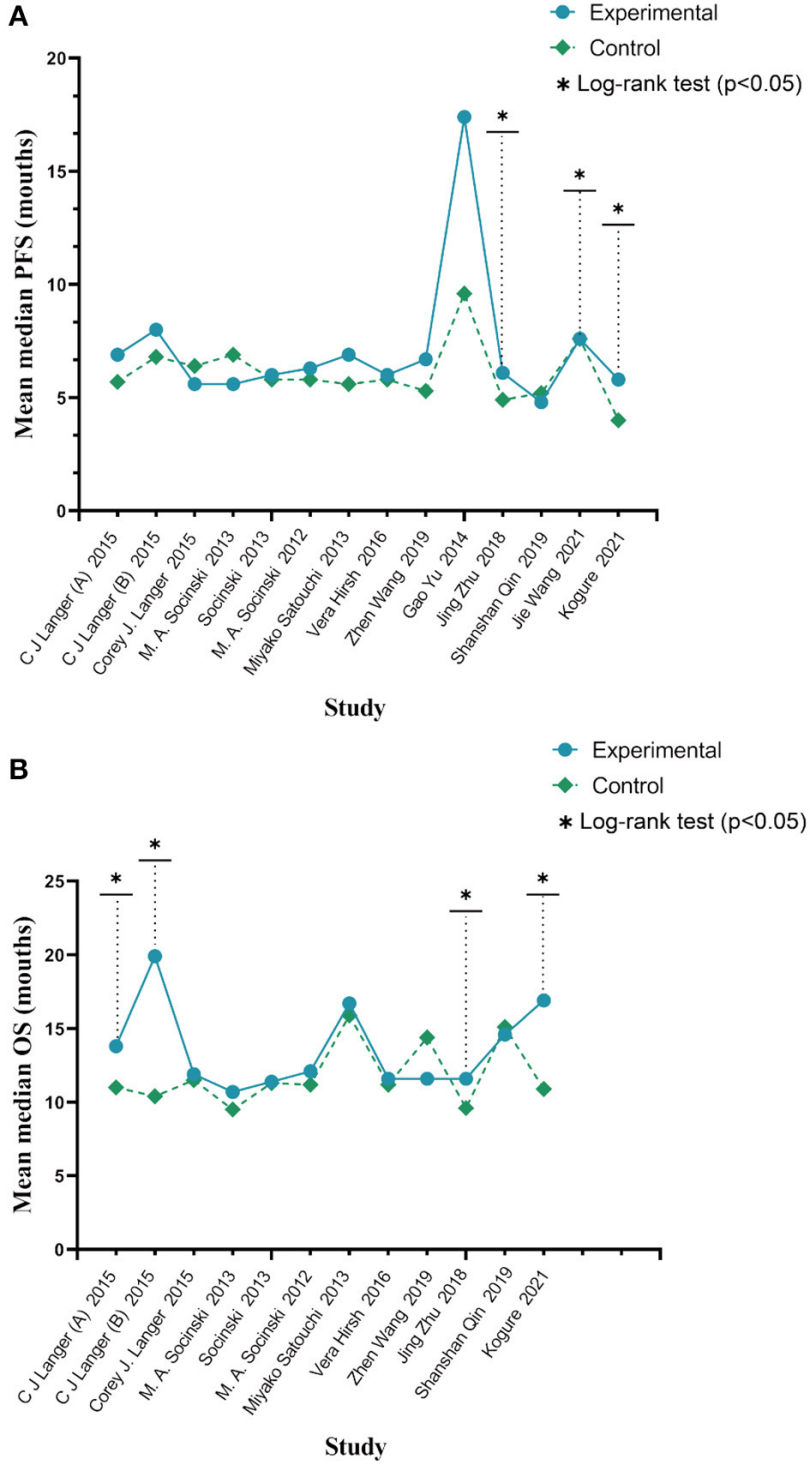
Mean median PFS time **(A)** and mean median OS **(B)**.

Based on a combined analysis of 13 studies, indicating that pooled PFS was of significant heterogeneity (*I*^2^ = 51.4%, *p* = 0.01), this study used a random-effects model. The results (Figure 4A) demonstrated that the experimental group had more profit than the control group [HR: 0.84, 95% CI: (0.83, 0.94), *p* = 0.003]. By analyzing subgroups (Table 3), nab-P + C [HR: 0.83, 95% CI: (0.74, 0.94), *p* = 0.003], the age >70 years [HR: 0.62, 95% CI: (0.40, 0.97), *p* = 0.038], and SCC rate ≥ 65% [HR: 0.74, 95% CI: (0.56, 0.97), *p* = 0.03] decreased HR of PFS among all of PFS. Among nab-P + C (*I*^2^ = 57.9%, *p* = 0.008), the age >70 years (*I*^2^ = 81.3%, *p* = 0.005), and SCC rate ≥ 65% (*I*^2^ = 77.1%, *p* = 0.002), the PFS results were analyzed by 13 trials. Of these 13 trials, three were reported to be statistically significant, improving median PFS probability in the experimental group (*p* < 0.05). The median PFS time in nab-P + C/Cis arm ranged from 5.6 to 17.4 (mean 7.12) months, and in the control arm, it ranged from 4.9 to 9.6 (mean 6.10) months (Figure 5A).

The data of OS was provided in 11 articles. The pooled OS was of significant heterogeneity (*I*^2^ = 56.5%, *p* = 0.011). Hence, a random-effects model was also used. A comparison of the experimental and the control groups (Figure 4B) indicated that nab-P + C had prolonged OS [HR: 0.86, 95% CI: (0.78, 0.96), *p* = 0.008]. During the subgroup analysis (Table 3), considering nab-P + C [HR: 0.86, 95% CI: (0.77, 0.96), *p* = 0.008] and the age >70 years [HR: 0.63, 95% CI: (0.49, 0.81), *p* < 0.001], significant heterogeneity existed in nab-P + C (*I*^2^ = 60.5%, *p* = 0.007) and the age > 70 years (*I*^2^ = 55.5%, *p* = 0.106). The results of median OS were calculated in 12 trials, with 4 studies showing prolonged OS in the experimental arm (*p* < 0.05). The median OS time in the nab-P + C/Cis group was 10.7–19.9 (mean, 13.57) months in comparison to 9.5–15.9 (mean, 11.83) months in the control group (Figure 5B).

#### 3.1.3. Security analysis

**Figure 6:**
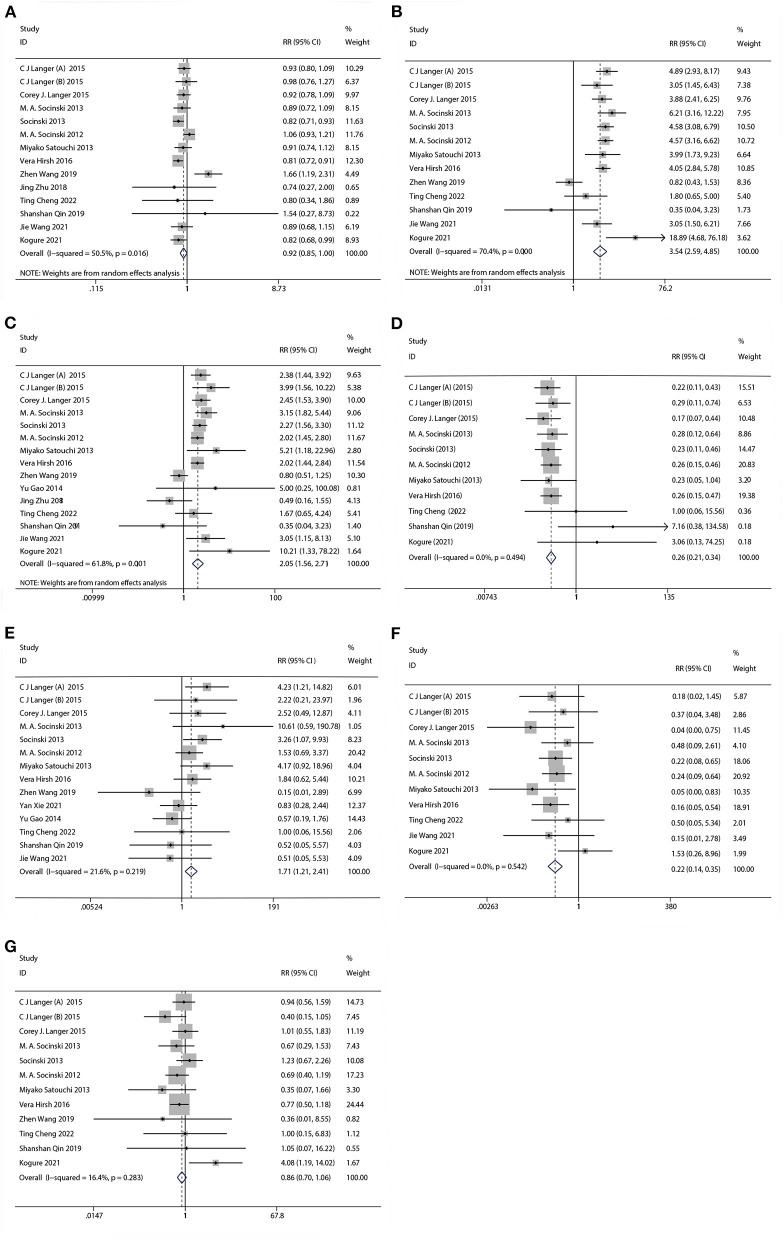
Forest plots of advance events for nab-P + C/Cis in the therapeutic of NSCLC. **(A)** Neutropenia, **(B)** anemia, **(C)** thrombocytopenia, **(D)** neuropathy, **(E)** gastrointestinal reactions, **(F)** arthralgia/myalgia, and **(G)** fatigue.

The results of treatment associated AEs grade ≥ 3 are shown in Table 4 and Figure 6. AEs can be divided into hematologic and non-hematologic AEs, with neutropenia [control arms vs. experiment arms (51%: 47%)] being the most common as shown in Table 5. The pooled result of fatigue [RR: 0.86, 95% CI: (0.70, 1.06), *p* = 0.155] showed no significant differences between the experimental and the control groups. When compared to the control group, the therapy with nab-P + C/Cis could abate the occurrence rate of neutropenia [RR: 0.92, 95% CI: (0.85, 0.99), *p* = 0.048], neuropathy [RR: 0.26, 95% CI: (0.21, 0.34), *p* < 0.001], and arthralgia/myalgia [RR: 0.22, 95% CI: (0.04, 0.19), *p* < 0.001]. Furthermore, the incidence of anemia [RR: 3.54, 95% CI: (2.59, 4.85), *p* < 0.001], thrombocytopenia [RR: 2.05, 95% CI: (1.56, 2.71), *p* < 0.001], and gastrointestinal reactions [RR: 1.71, 95% CI: (1.21, 2.41), *p* < 0.001] were higher in the therapy based on nab-P. Among AEs, the case of neutropenia (*I*^2^ = 50.5%, *p* = 0.016), anemia (*I*^2^ = 70.4%, *p* = 0.000), and thrombocytopenia (*I*^2^ = 61.8%, *p* = 0.001) showed significant heterogeneity. Meanwhile, this study showed that there was no significant heterogeneity in the event of neuropathy (*I*^2^ = 0.0%, *p* = 0.494), gastrointestinal reactions (*I*^2^ = 21.6%, *p* = 0.219), arthralgia/myalgia (*I*^2^ = 0.0%, *p* = 0.542), and fatigue (*I*^2^ = 16.4%, *p* = 0.283).

**Table 4:** The meta-analysis result of the adverse events in comparative studies.

**Adverse events**	***N***	**Incidence over sample size**		**RR**	**95% CI**	***I*^2^, *p* heterogeneity**	***p*-value**
**Hematologic**		**Experimental**	**Control**				
Neutropenia	14	1,293/2,769	1,467/2,850	0.92	0.85, 0.99	*I*^2^ = 50.5%, *P* = 0.016	0.048
Anemia	13	735/2,717	186/2,766	3.54	2.59, 4.85	*I*^2^ = 70.4%, *P* = 0.000	0.000
Thrombocytopenia	15	498/2,809	241/2,857	2.05	1.56, 2.71	*I*^2^ = 61.8%, *P* = 0.001	0.000
**Non-hematologic**						
Neuropathy	11	71/2,548	278/2,593	0.26	0.21, 0.34	*I*^2^ = 0.0%, *P* = 0.494	0.000
Gastrointestinal reactions	14	81/2,691	48/2,741	1.71	1.21, 2.41	*I*^2^ = 21.6%, *P* = 0.219	0.002
Arthralgia/myalgia	11	20/2,620	99/2,670	0.22	0.14, 0.35	*I*^2^ = 0.0%, *P* = 0.542	0.000
Fatigue	12	151/2,607	180/2,495	0.86	0.70, 1.06	*I*^2^ = 16.4%, *P* = 0.283	0.155

**Table 5:** The profile of adverse events in various studies.

**References**	**Intervention**		**Neutropenia**		**Anemia**		**Thrombocytopenia**		**Neuropathy**		**Gastrointestinal reactions**		**Arthralgia/myalgia**		**Fatigue**	
	**E**	**C**	**E**	**C**	**E**	**C**	**E**	**C**	**E**	**C**	**E**	**C**	**E**	**C**	**E**	**C**
Langer et al. (A) (13)	nab-P + C	sb-P + C	134	152	74	16	45	20	9	44	12	3	1	6	24	27
Langer et al. (B) (13)	nab-P + C	sb-P + C	39	51	22	8	18	5	5	19	2	1	1	3	5	14
Langer et al. (14)	nab-P + C	sb-P + C	136	147	74	19	54	22	5	29	5	2	0	11	20	20
Socinski et al. (15)	nab-P + C	sb-P + C	95	103	58	9	49	15	7	24	5	0	2	4	9	13
Socinski et al. (16)	nab-P + C	sb-P + C	198	244	123	27	79	35	9	40	13	4	4	18	22	18
Socinski et al. (17)	nab-P + C	sb-P + C	242	244	139	31	93	47	15	58	15	10	5	21	21	31
Satouchi et al. (18)	nab-P + C	sb-P + C	49	56	23	6	10	2	2	9	8	2	0	10	2	6
Hirsh et al. (19)	nab-P + C	sb-P + C	227	286	135	34	87	44	14	54	9	5	3	19	33	44
Wang et al. (20)	nab-P + C	Gem + C	42	27	13	17	21	28	0	0	0	3	0	0	0	1
Xie and Wang (21)	nab-P + Cis	Gem + Cis	0	0	0	0	0	0	0	0	5	6	0	0	0	0
Gao and Zhu (22)	nab-P + Cis	Pa + Cis	0	0	0	0	2	0	0	0	4	7	0	0	0	0
Zhu et al. (23)	nab-P + Cis	Gem + Cis	6	8	0	0	4	8	0	0	0	0	0	0	0	0
Chen and Sun (24)	nab-P + C	Pa + C	8	10	9	5	10	6	1	1	1	1	1	2	2	2
Qin et al. (25)	nab-P + Cis	Gem + Cis	3	2	1	3	1	3	3	3	1	2	0	0	1	1
Wang et al. (26)	nab-P + C + tis	Pa + C + tis	54	62	27	9	15	5	0	0	1	2	0	3	0	0
Kogure et al. (27)	nab-P + C	Doc	60	75	37	2	10	1	1	0	0	0	3	2	12	3
Event (*n*, %)	-	-	1,293	1,467	735	186	498	241	71	281	81	48	20	99	151	180
			47%	51%	27%	6.7%	18%	8%	2.7%	11%	3%	1.8%	0.8%	3.7%	5.8%	7.2%
nab-P + C (*n*, %)	-	-	48%	-	27%	-	18%	-	2.7%	-	2.7%	-	0.7%	-	5.8%	-
nab-P + Cis (*n*, %)	-	-	8.7%	-	7.1%	-	5.3%	-	7.1%	-	9.8%	-	0%	-	2.4%	-
Total	-	-	2,769	2,850	2,717	2,766	2,809	2,857	2,548	2,593	2,691	2,741	2,620	2,670	2,607	2,495

nab-p, nab-paclitaxel; C, carboplatin; Cis, cisplatin; Gem, gemcitabine; sb-P, solvent-based paclitaxel; Pa, paclitaxel; P-Lip, paclitaxel liposome; Doc, docetaxel; tis, tislelizumab; NR, not reported.

#### 3.1.4. Meta-regression analysis

**Figure 7:**
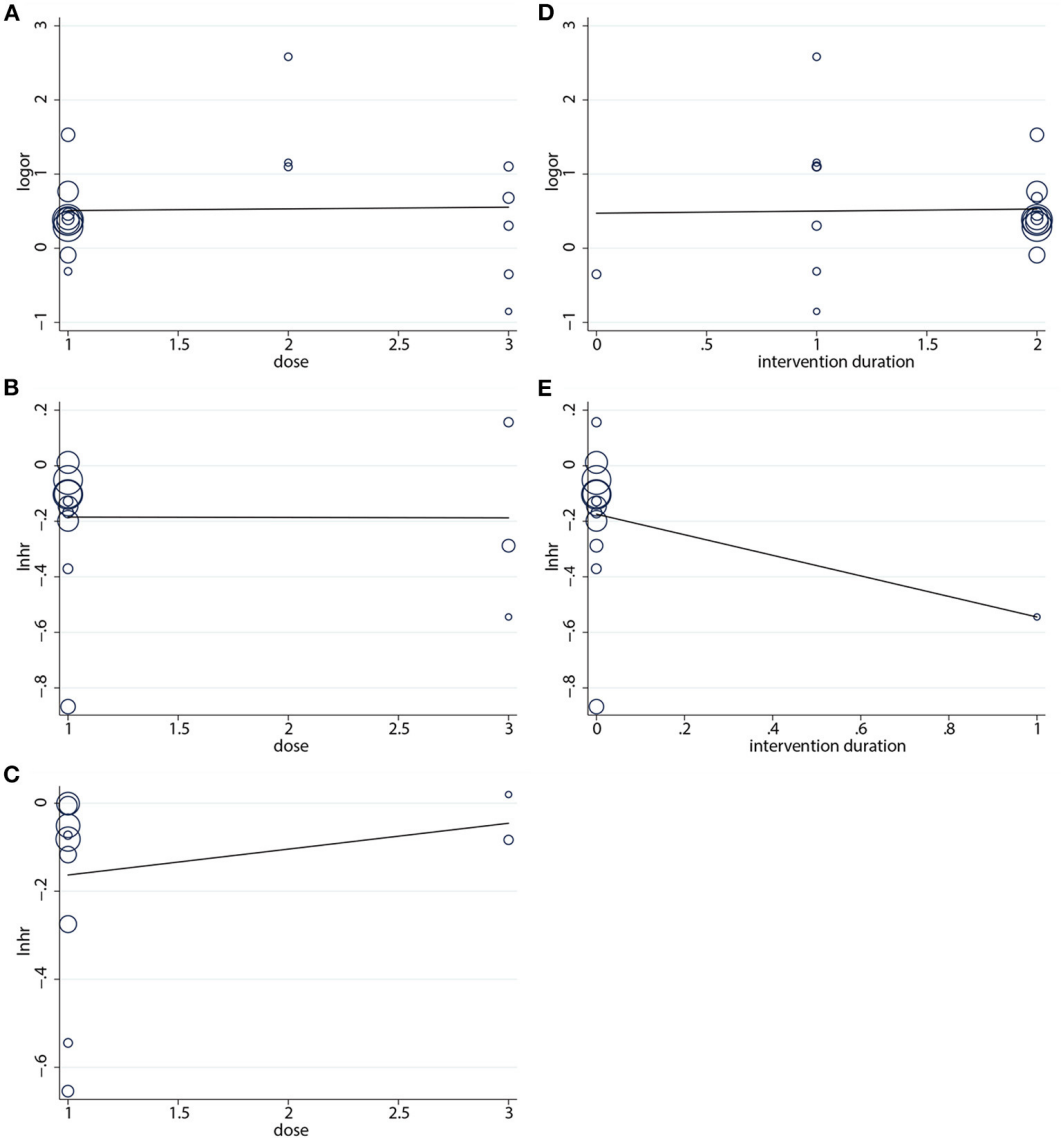
Regression analysis of **(A)** ORR, **(B)** PFS, **(C)** OS on intervention dose, **(D)** ORR, and **(E)** PFS on intervention duration.

The aim of this analysis was to appraise the correlation by dose and duration of the intervention (months) of nab-P + C/Cis with ORR, PFS, and OS. According to the results, no linear correlation was observed for the absolute changes in these factors with the intervention dose and intervention duration (Figure 7). Based on the data of intervention, the duration of OS was consistent across the 11 studies, and thus no results were available in OS.

#### 3.1.5. Sensitivity analyses

**Figure 8:**
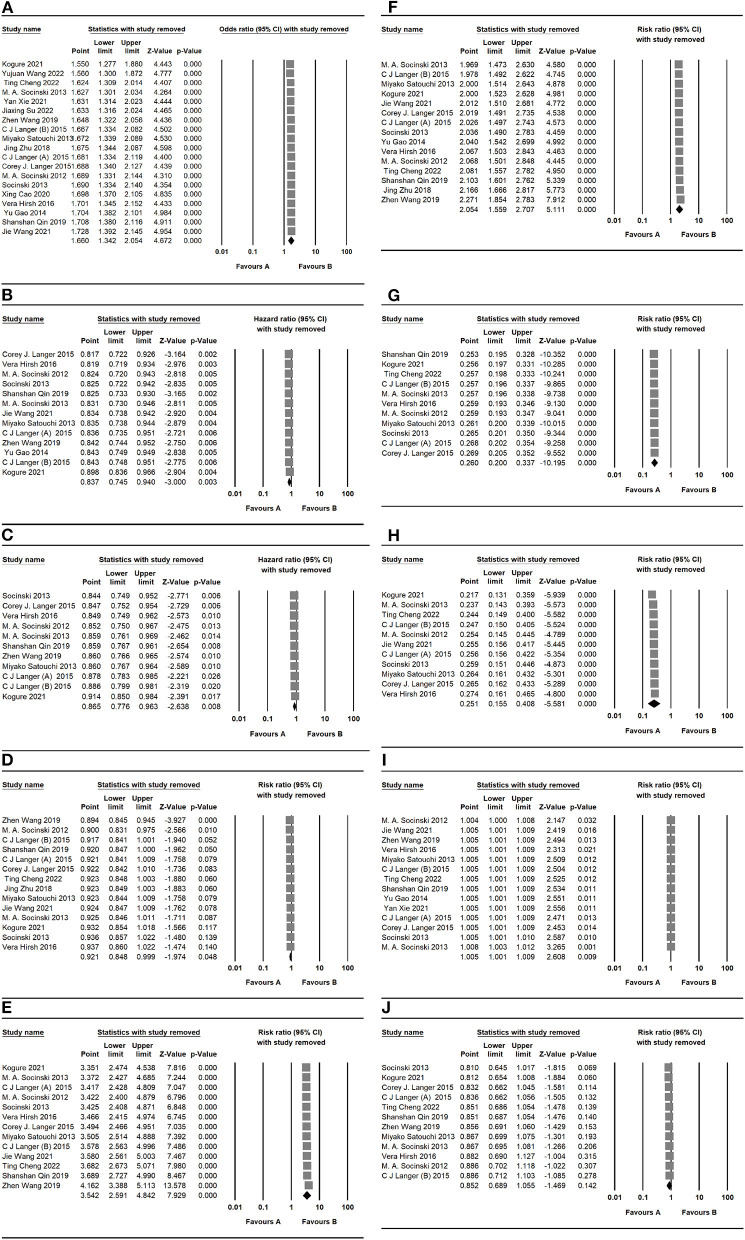
Sensitivity analysis of primary outcomes, including **(A)** ORR, **(B)** PFS, **(C)** OS, **(D)** neutropenia, **(E)** anemia, **(F)** thrombocytopenia, **(G)** neuropathy, **(H)** gastrointestinal reactions, **(I)** arthralgia/myalgia, and **(J)** fatigue for nab-P + C/Cis in the therapeutic of NSCLC. Favors A is the experimental and Favors B is the control.

We used the sensitivity analysis to evaluate the outcomes. These outcomes were presented with no significant modifications of ORR, PFS, OS, and AES (Figure 8) after deleting the studies one by one, suggesting that the valid results of therapeutic response were relatively stable in patients under nab-P + C/Cis treatment.

#### 3.1.6. Assessment of publication bias

**Figure 9:**
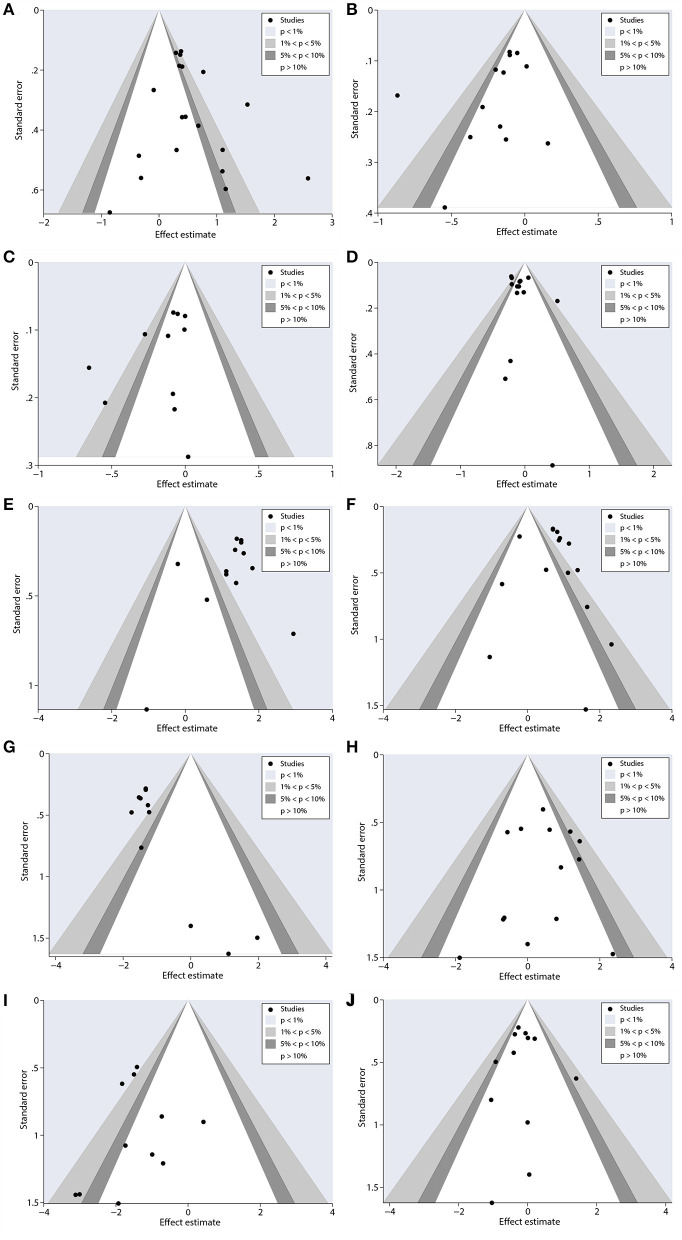
Contour-enhanced funnel plots to analyze potential publication bias. **(A)** ORR, **(B)** PFS, **(C)** OS, **(D)** neutropenia, **(E)** anemia, **(F)** thrombocytopenia, **(G)** neuropathy, **(H)** gastrointestinal reactions, **(I)** arthralgia/myalgia, and **(J)** fatigue.

Contour-enhanced funnel plots were performed to appraise the results of potential publication bias. Conventional assignment criteria at the statistical significance level (*p* < 0.01, < 0.05, and < 0.1) were added to the funnel plot for distinguishing the detailed causes of publication bias. Figure 9 shows that there were asymmetrical, with many missing studies that fall in the areas of high statistical significance. Using Begg's and Egger's tests for further assessments, the results obtained were quantifiable, indicating the absence of potential publication bias. More details are presented in Table 6. In total, this study illustrated that the asymmetry may occur as a result of reasons other than publication bias.

**Table 6:** Begg's and Egger's tests of the meta-analysis.

**Closing indicators**	**Begg (*p*)**	**Egger (*p*)**
ORR	0.484	0.332
PFS	0.127	0.183
OS	0.436	0.174
Neutropenia	0.584	0.398
Anemia	0.360	0.353
Thrombocytopenia	0.488	0.715
Neuropathy	0.062	0.010
Gastrointestinal reactions	0.743	0.777
Arthralgia/myalgia	0.276	0.771
Fatigue	0.945	0.942

#### 3.1.7. Quality of evidence

**Figure 10:**
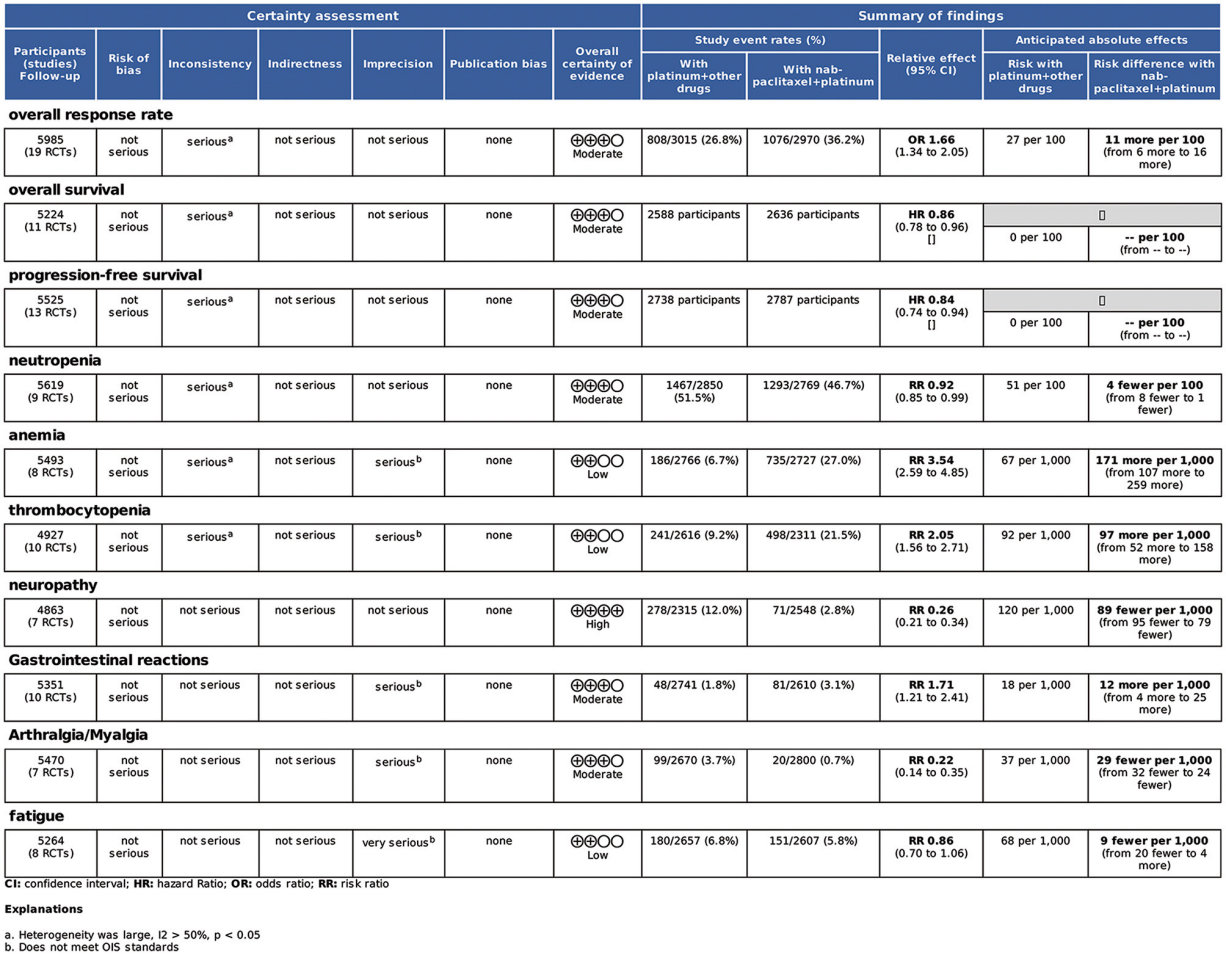
GRADE evidence profile for all outcome measures.

The quality of the evidence table (Figure 10) was assessed for each outcome. Neutropenia presented high evidence. ORR, PFS, OS, neuropathy, gastrointestinal reactions, and arthralgia/myalgia outcomes had moderate certainty. Anemia, thrombocytopenia, and fatigue presented with low certainty.

## 4. Discussion

The meta-analysis included 19 randomized clinical studies with 6,011 participants and presented the effectiveness of nab-P combined with carboplatin/cisplatin interventions in achieving improved ORR, prolonged PFS and OS, and declined AEs. These studies revealed that the combined regiments increase anticancer efficacy in patients with NSCLC and reasonable AEs occurrence which is generally acceptable.

Depending on the results, nab-P combined with platinum (carboplatin/cisplatin) elevated ORR [OR: 1.66, 95% CI: (1.34, 2.05)], and extended PFS [HR: 0.84, 95% CI: (0.74, 0.94)] and OS [HR: 0.86, 95% CI: (0.78, 0.96)], which indicated that nab-P + C/Cis was more effective than carboplatin/cisplatin in combination with conventional chemotherapeutic agents or traditional paclitaxel. For subgroup analysis based on interventions, first, only a few studies (22, 25) could obtain HR for PFS and OS from the original studies of nab-P + Cis interventions for treatment. Next, the nab-P + Cis intervention was not statistically significant in ORR [OR: 0.87, 95% CI: (0.44, 1.72)]. In this study, we did not obtain information about which combination chemotherapy regimen was more beneficial for patients with NSCLC between nab-P + C and nab-P + Cis. The lack of directly comparable clinical trials prevents us from determining which chemotherapy regimen is more effective. In an analysis of subgroups according to median age, the nab-P + C/Cis regimen significantly increased ORR and extended the duration of PFS and OS in the age > 70 years arm. Furthermore, by subgroup analysis depending on the SCC rate, the nab-P + C/Cis regimen was found to enhance ORR and growth in PFS greater in the SCC rate ≥ 65% arm. This implies that nab-P + C/Cis chemotherapy regimens may benefit more dramatically in older patients with NSCLC and patients with squamous cell carcinoma.

AEs can be separated into hematologic and non-hematologic events, based on the occurrence of neutropenia with the highest rate. Among the adverse results (grade ≥ 3) that showed in terms of hematological toxicities, when compared to carboplatin or cisplatin in combination with conventional chemotherapy agents or traditional paclitaxel, anemia [3.54 (2.59, 4.85)] and thrombocytopenia [2.05 (1.56, 2.71)] markedly increased in patients who were treated for nab-P + C/Cis, and neutropenia [0.92 (0.85, 0.99)] was relatively lower. In relation to non-hematological toxicities, neuropathy [0.26 (0.21, 0.34)] and arthralgia/myalgia [0.22 (0.14, 0.35)] occurred less in the nab-P + C group. However, significantly more incidence of gastrointestinal reactions [1.71 (1.21, 2.41)] were observed in nab-P + C/Cis compared with carboplatin/cisplatin in combination with conventional chemotherapy agents or traditional paclitaxel. Moreover, fatigue [0.86 (0.70, 1.06), *p* = 0.155] did now show a statistically significant difference.

In a present meta-analysis of the efficacy and safety of nab-P in combination with carboplatin for NSCLC, Tan et al. (31) demonstrated that, in comparison to control, nab-P + C improved ORR and extended PFS and OS. In the area of AEs, nab-P + C raised the incidence of anemia (grade ≥ 3) and diminished the risk of grade ≥ 3 neuropathy and arthralgia. There was no previous meta-analysis of nab-P + Cis intervention in NSCLC, but a review analysis (32) has reported an aggressive effect of nab-P + Cis intervention in NSCLC, with a tendency to obtain a higher ORR and improved PFS and OS. nab-P + Cis was more well-tolerated as illustrated in the trial by Hattori et al. (33). Our findings were similar to those that have been reported.

Patients significantly benefit in terms of ORR after nab-P + C/Cis chemotherapy (12, 17). The mechanism of action of albumin combined with carboplatin/cisplatin for intervention against NSCLC may be to utilize albumin features for increasing the antitumor role of the drug. Tumors may be fed by albumin as a nutritive substance in the tumor microenvironment and it possibly promotes tumor growth (34). Nab-P, an albumin-bound drug, leverages these mechanisms to enhance the delivery specifically to tumors that an affinity for albumin. The gp60 receptor-specific endothelial cells activate transmembrane transport, and albumin accumulates in the tumor environment by the EPR effect to reach tumors (8, 10, 11). The studies show that the coadministration of albumin-bound paclitaxel with gemcitabine enhances the gemcitabine levels in tumors of a mouse model of pancreatic cancer (35, 36). This supports the possibility that albumin-bound paclitaxel could increase the level of combined drugs reaching the tumor, but more research data are required to support this.

This meta-analysis included clinical studies of nab-P in combination with cisplatin, and it had a higher total sample size. Second, contour-enhanced funnel plots and Begg's and Egger's tests demonstrated that no evidence of publication bias was observed in this study. Besides, outcome quality was graded according to grade guidelines, and correlations for ORR, PFS, and OS with intervention dose and intervention duration were estimated using a regression analysis. However, there are several limitations to this analysis. First, the study shows considerable heterogeneity with respect to ORR, PFS, OS, neutropenia, anemia, and thrombocytopenia. Although using the subgroup analysis and a random-effects model, there is no way to decrease heterogeneity. In the second place, with two of the accepted articles, no HR was provided and calculated using Kaplan–Meier survival curves, which may cause a potential risk of bias (22, 26). Finally, most of the studies of nab-P + Cis in terms of primary data are unavailable or unable to calculate HR for PFS and OS, and AEs are not graded.

## 5. Conclusion

In general, this meta-analysis demonstrates that nab-P combined with carboplatin/cisplatin in patients with NSCLC could significantly increase ORR and prolong PFS and OS. However, PFS and OS do not show any particularly visible benefit; therefore, a greater number of studies will be demanded to explore further. Based on the efficacy and tolerability of nab-P combined with carboplatin/cisplatin, it may provide an economically disadvantaged patient with an affordable treatment option.

## Data availability statement

The original contributions presented in the study are included in the article/supplementary material, further inquiries can be directed to the corresponding authors.

## Author contributions

TT and SL were responsible for drafting the initial text and making equivalent revisions. WH and TY conducted data analysis for the revised manuscript, while QZ and XZ extracted and analyzed data from the original manuscript. XC, XZ, and TX played significant roles in the conceptualization and design of the study. All authors contributed to the article and approved the submitted version.
